# Residual Chlorella-Based Cellulose Nanofibers and Their Quaternization Modification and Efficient Anionic Dye Adsorption

**DOI:** 10.3390/ma16103642

**Published:** 2023-05-10

**Authors:** Lina Zhang, Xiaomin Huo, Jin Zhu, Changbin Liu, Lianfeng Wang

**Affiliations:** 1 School of Materials Science and Engineering, Dalian Jiaotong University, Dalian 116028, China; 2 Dalian Product Quality Inspection and Testing Institute Co., Ltd., Dalian 116021, China; 3 School of Biological Engineering, Dalian Polytechnic University, Dalian 116034, China

**Keywords:** Chlorella residue, nanofiber of cellulose, quaternization, dye adsorption

## Abstract

Dye is an anionic common pollutant in industrial wastewater and poses a great threat to the environment and human health. Owing to its advantageous adsorption capacity, nanocellulose is widely used for wastewater treatment. The cell walls of Chlorella mainly comprise cellulose instead of lignin. In this study, residual Chlorella-based cellulose nanofiber (CNF) and cationic cellulose nanofiber (CCNF) with surface quaternization were prepared through homogenization. Moreover, Congo red (CR) was used as a model dye to measure the adsorption capacity of CNF and CCNF. The adsorption capacity was almost saturated when CNF and CCNF contacted CR for 100 min, and the adsorption kinetics coincided with the pseudo-secondary kinetics model. The initial concentration of CR considerably affected its adsorption on CNF and CCNF. Below the initial concentration of 40 mg/g, the adsorption on CNF and CCNF considerably increased with the increase in the initial concentration of CR. Based on the sorption isotherms analysis of CNF and CCNF, the Langmuir model fitted best with the experimental data. Thus, CNF and CCNF surfaces were uniform, and monolayer adsorption occurred. The adsorption of CR on CNF and CCNF was greatly affected by the pH value, and the acidic medium favored the adsorption of CR (especially for CCNF). CCNF showed a more advantageous adsorption capacity, with a maximum value of 1657.89 mg/g, compared to that of CNF (190.0 mg/g). According to the findings of this study, residual Chlorella-based CCNF could be a very promising adsorbent candidate for removing anionic dyes from wastewater.

## 1. Introduction

Cellulose is a major component of plant cell walls and one of the most abundant substances on Earth. Photosynthesis allows plants to produce billions of tons of cellulose, making them an inexhaustible and renewable resource [1,2]. The ample hydroxyl and carboxylic groups of cellulose can be modified or grafted with organic moieties, offering extra functional groups that are customizable for specific applications. Cellulose can be prepared in several derivatives, including nanomaterials such as cellulose nanocrystals (CNCs), cellulose nanofibrils (CNFs), and TEMPO (2,2,6,6-tetramethylpiperidine-1-oxyl radical)-mediated oxidized cellulose nanofibrils (TOCNFs). Cellulose nanofiber (CNF) is cellulose on a nanoscale. Owing to its large specific surface area, good biocompatibility, and surface activity, it has been extensively used in preparing nanocomposites [3], 3D printing materials [4], packaging materials [5], electronic energy-storage materials [6], water treatment [7], and biomedical fields [8]. 

CNF is mainly prepared from agricultural byproducts (including wood, cotton, wheat straw, and palm) through various methods (including acid hydrolysis, tempo-mediated oxidation, mechanical breakdown, and enzyme-assisted hydrolysis) [9,10,11]. However, for the industrial production of CNFs from these materials, pretreatment processes with great energy consumption are usually necessary, such as mechanical mincing, degumming, and pulping [12]. Furthermore, a large number of land resources are needed to support the growth of these materials. Unlike these agriculture byproducts, microalgae are single-celled and have a simple cellular structure. Their cell walls mainly comprise cellulose instead of lignin, which facilitates an easier purification of nanofibers [13,14,15]. Additionally, microalgae have simple requirements for growth, do not occupy farmlands, exhibit high-yield rapid growth in a short growth period, and could be mass cultivated throughout the year [16]. Cellulose is abundantly present in the solid waste of microalgae (including *Chlorella* and *Nannochloropsis*), generated after refining lipids and bioactive components. This solid waste is often used to produce low-value products, including animal feed, fertilizers, and methane gas [17,18,19]. The economic value of this cellulosic waste would be further increased if utilized for CNF preparation. 

Dye is an indispensable material in the textile and printing industries [20]. Several thousand tons of dyes are discarded directly into the water every year in the textile industry alone [21,22]. Being a complex organic molecule, dye is toxic and has poor biodegradability, and its access to water poses a great threat to the environment and human health [23,24]. Presently, dyes in wastewater are removed through various approaches, including coagulation/flocculation [25], ion exchange [26], membrane separation [27], adsorption [28,29], advanced oxidation [27], photocatalytic degradation [30], and biological elimination [31]. Specifically, the adsorption technique is an economic, effective, and sustainable strategy. CNF has the characteristics of a large aspect ratio, specific surface area, high mechanical strength, and low weight. Additionally, its surface is enriched with hydroxyl groups, and it could act as a desirable adsorbent [32]. In the past decades, cellulose has been used as an adsorbent for various pollutants [33,34,35]; however, its application as an effective adsorbent is impeded because it lacks active functional groups [24].

In this study, CNF was prepared from residual Chlorella without energy-intensive pretreatment, which can effectively improve the economic value of Chlorella and save energy consumption. Further, anhydrous glyceryl trimethyl ammonium chloride (GTMAC, C_6_H_14_ClNO) was used for modification to generate cationic CNF (CCNF) with surface quaternization. We evaluated the adsorption efficacy of residual Chlorella-based CNF and CCNF using Congo red (CR, C_32_H_22_N_6_Na_2_O_6_S_2_) as a model dye. The results show that CCNF has very good anionic dye adsorption properties and can be used as a promising environmentally friendly adsorbent candidate for removing anionic dyes from industrial wastewater.

## 2. Materials and Methods

### 2.1. Materials and Chemicals

The materials used for CNF preparation were residues of laboratory Chlorella after lipid extraction. Chemicals, including experimental anhydrous GTMAC (C_6_H_14_ClNO, >95%), sodium hydroxide (NaOH, 97%), sodium acetate buffer solution (pH 5.2), sodium chlorite (NaClO_2_, 80%), and CR (C_32_H_22_N_6_Na_2_O_6_S_2_, >98%), were purchased from Shanghai Aladdin Bio-Chem Technology Co., LTD, Shanghai, China. *Chlorella* powder was obtained from Shanghai Guangyu Biological Technology Co., Ltd. (Shanghai, China).

### 2.2. Methods of Experiments

#### 2.2.1. Preparation of CNF and CCNF

The methods for preparing CNF and CCNF were based on those of Ahaby et al. [15] and Aihua Pei et al. [32]. Detailed procedures are as follows: 5 g of Chlorella residue was weighed and mixed with double-distilled water (double-distilled water, 150 mL). The mixture was placed in a water bath at 90 °C for 1 h. The obtained sediment was combined with 200 mL sodium hydroxide (NaOH) solution (5% *w*/*v*), which was then subjected to a water bath with continuous stirring (150 rpm) at 80 °C for 2 h. Afterward, the mixture was centrifuged (10,000 rpm at 25 °C for 10 min), and the collected sediment was washed with double-distilled water until its pH became neutral. The sediment was mixed with sodium acetate buffer (100 mL) and sodium chlorite solution (100 mL). After continuous stirring (150 rpm) in a water bath at 80 °C for 1 h, the mixture was centrifuged again, and the collected sediment was washed with double-distilled water until its pH became neutral. Half of the obtained sediment was combined with NaOH solution (5% *w*/*v*), followed by the gradual addition of anhydrous GTMAC to the mixture during continuous stirring until the final concentration reached 1.32 mmol/g. The reaction system was incubated at 65 °C for 8 h. After neutralizing it with hydrochloric acid, the mixture was centrifuged, and the obtained sediment was washed thoroughly with double-distilled water. Subsequently, the sediment was mixed in deionized water, followed by stirring at 600 rpm for 24 h. The cellulose suspensions with and without quaternization modification were put into a high-pressure homogenizer. They were treated under a pressure of 40 MPa for 20 min to generate CNF and CCNF suspensions. The colloidal solutions of CNF and CCNF (10 mL) were transferred and weighed after freeze-drying, and their concentrations were detected.

#### 2.2.2. CNF Characterization

A scanning electron microscope (SEM, JSM-7800F, Akishima, Japan) and atomic force microscope (AFM, Bruker Dimension Icon, Billerica, MA, USA) were used for observing CNF and CCNF. A Fourier transform infrared spectrometer (FTIR, PerkinElmer Spectrum two, Waltham, MA, USA) and X-ray diffractometer were used for analyzing the IR spectra and X-ray diffraction (XRD, SHIMADZU XRD-7000, Kyoto, Japan), respectively, of the nanofibers. The scanning rate of the X-ray was 4 °/min, 2θ ranged from 10° to 40°, and the scanning step was 0.01°. The Segal method was used to calculate the crystallinity index (*CrI*), which is as follows: $CrI=\frac{I_{002}-I_{a}}{I_{002}}\times 100$, where *I*_002_ and *I*_*a*_ are the peak intensities of crystalline and amorphous cellulose, respectively.

#### 2.2.3. Calibration Curve Establishment for the Ultraviolet (UV) Absorption of CR with Varying Concentrations

To prepare CR solutions with varying concentrations, 600 mg of CR was weighed. A UV-visible (UV-Vis, PerkinElmer Lambda 35) spectrometer was used to measure the absorbance of the varying concentrations of CR at 497 nm. The concentration was adjusted to make its absorbance lie within the range of 0.1–1. The calibration curves of CR were fitted based on the Beer–Lambert law.

#### 2.2.4. Investigation on the Adsorption on CNF and CCNF of CR

Adsorption kinetics was performed as follows: (1) CNF suspension (0.1 g) and CCNF suspension (0.1 g) were collected at room temperature and transferred to respective flasks containing 100 mL CR dye solution (6 mg/L) and stirred at 250 rpm; (2) solutions of 2 mL were collected from each flask and centrifuged after treatment with CR dye for 1 min, 20 min, 40 min, 60 min, 80 min, 100 min, 120 min, 140 min, and 160 min, respectively; (3) the absorbance of the supernatant was measured using a UV-Vis spectrometer, and the concentration of CR in the remaining solution was calculated. Equation (1) was used to calculate the quantity of adsorption (*Q_t_*) on CNF and CCNF per unit mass after treatment for different durations.

(1) $$Q_{t}=\frac{\left(C_{0}-C_{t}\right)V}{m}$$

In this equation, *C*_0_ (mg/L) is the initial concentration of the dye solutions, *C_t_* (mg/L) is the dye concentration after adsorption, *m* (g) is the mass of the adsorbents, and *V* (L) is the volume of dye solutions subjected to adsorption.

The fitting of experimental values was performed using the pseudo-first-order Equation (2) and pseudo-second-order kinetics Equation (3) [24].

(2) $$\log\left(Q_{e}-Q_{t}\right)=\;\log Q_{e}-\frac{K_{1}}{2.303}t$$

(3) $$\frac{t}{Q_{t}}=\frac{1}{K_{2}Q_{e}^{2}}+\frac{t}{Q_{e}}$$

In Equations (2) and (3), *Q_e_* is the amount of dye adsorbed by adsorbents upon equilibrium, *t* is adsorption time, and *K*_1_ and *K*_2_ are rate constants.

The surface charge is an important index with which to study the adsorption of materials. The point of zero charge is determined by potentiometric titration according to the procedure that is reported by El-kordy et al. [36]. Additionally, the effect of pH on CR adsorption was evaluated according to the following steps: CNF (0.1 g) and CCNF (0.1 g) were added to respective 50 mL dye solutions; the initial pH of the dye solutions was adjusted, using HCl and NaOH solutions, to 3, 4, 5, 6, 7, 8, 9, and 10, respectively; adsorption was performed by stirring at 250 rpm for 100 min at room temperature, followed by centrifugation (10,000 rpm at 25 °C for 5 min). The concentration of CR in the supernatant was measured using a UV-Vis spectrometer (PerkinElmer Lambda-35). The amounts of CR adsorption at varying pH values were calculated, and the effect of the initial pH values of dye solutions on the adsorption capacity of CNF and CCNF were analyzed. 

The effect of initial dye concentration on the adsorption capacity of CNF and CCNF was analyzed as follows: CNF (0.1 g) and CCNF (0.1 g) were added to 50 mL CR solutions with different initial concentrations, respectively; the mixture was centrifuged after adsorption induced by stirring at 250 rpm for 100 min at room temperature; a UV-Vis spectrometer was used to detect the dye concentration in the supernatant. The amount of adsorption was calculated, based on which the effect of the initial concentration of CR on the adsorption capacity of CNF and CCNF was evaluated. The Langmuir and Freundlich isotherm model equations were used for the fitting of adsorption data [24]. The isotherm equations are reflected as Equations (4) and (5).

(4) $$Q_{e}=\frac{K_{L}\;\cdot \;Q_{m\;}\;\cdot \;C_{e}}{1+\left(K_{L}\;\cdot \;C_{e}\right)}$$

In this equation, *Q_e_* (mg/g) is the amount of dye adsorbed per unit mass of adsorbents, *C_e_* (mg/L) is the equilibrium concentration, *K_L_* (L/mg) is the Langmuir isotherm constant, and *Q_m_* (mg/g) is the maximum adsorption capacity.

(5) $$Q_{e}=K_{f}\;\cdot \;C_{e}^{\frac{1}{n}}$$

In this equation, *K_f_* represents the Freundlich constant, and n denotes adsorption intensity. 

## 3. Results and Discussion

### 3.1. Characterization of CNF and CCNF

**Figure 1:**
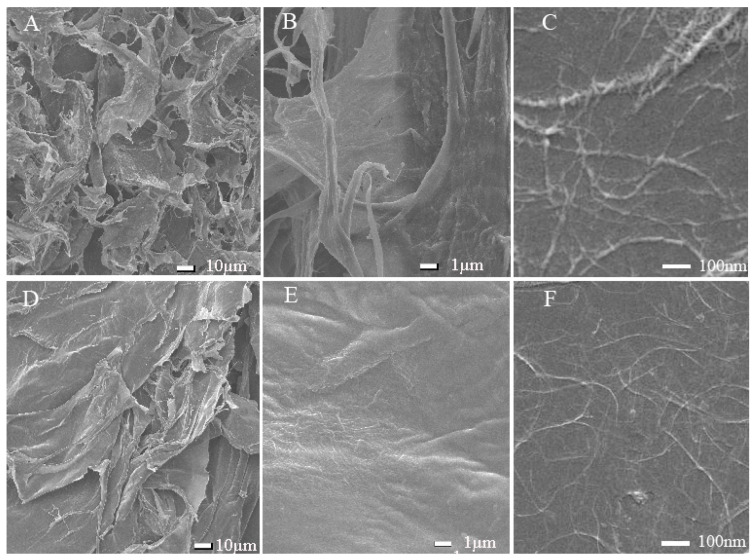
Scanning electron microscopic (SEM) images of CNF (**A**–**C**) and CCNF (**D**–**F**).

**Figure 2:**
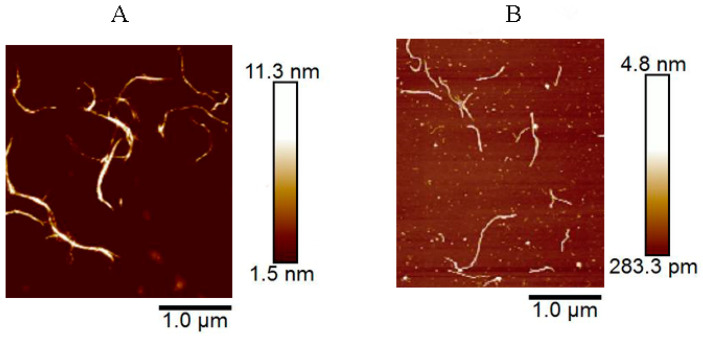
Atomic force microscopic images (AFM) of CNF (**A**) and CCNF (**B**).

The prepared CNF and CCNF suspensions were freeze-dried and weighed, and the obtained CNF and CCNF concentrations were calculated to be 1.58% and 1.06% (*w*/*v*), respectively. SEM was used for the observation of samples, and the results are illustrated in Figure 1. At the scale of both 10 µm and 1 µm, lamellar structures were observed for CNF (A) and CCNF (E). The lamellar structures of CNF intertwined with each other, creating what appeared to be a honeycomb-like structure. At the scale of 1 µm, both CNF and CCNF of the lamellar structure were found to consist of intertwined small fibrils. At the scanning scale of 100 nm, it could be seen that the nanoscale linear fibers intertwined compactly with each other; however, compared to CNF, the fibrils of CCNF were finer and smaller, which presented a denser lamellar structure. To further characterize the morphological features of CNF and CCNF, their prepared suspensions were diluted and characterized using AFM. The results are shown in Figure 2. Dispersed nanofibers were observed, as shown in the images. The average diameter of CNF was 4.1 ± 2.3 nm, whereas its average length was 375 ± 35.3 nm. However, CCNF was finer, shorter, and smaller. Hence, nanofibers were successfully prepared from the waste of Chlorella residues. 

**Figure 3:**
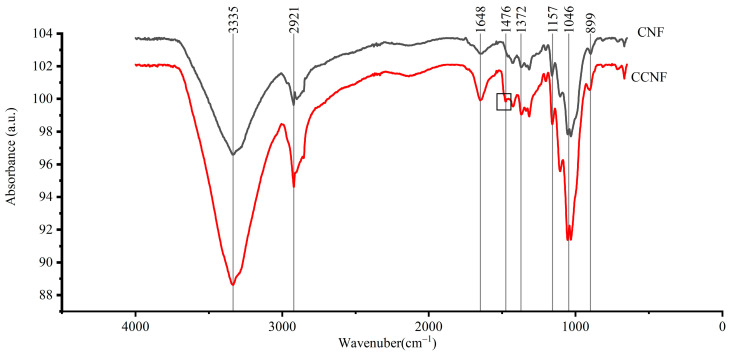
Fourier transform infrared spectrometer (FTIR) spectrum of cellulose nanofiber (black) and CCNF (red).

IR spectra analysis was performed for CNF and CCNF samples using an FTIR. The results are shown in Figure 3. The band located at 3335 cm^−1^ was attributed to the stretching vibration of intermolecular hydroxyl groups of cellulose [2,37]. The band located at 2921 cm^−1^ corresponded to the stretching vibration of the C-H bond [38]. A band at 1157 cm^−1^ corresponded to the asymmetric stretching of β-glucose ether bonds (C-O-C) in cellulose [39]. At 1648 cm^−1^, the bending vibration and stretching were induced by water absorption on CNF and CCNF [32]. The bands at 1425 cm^−1^, 1372 cm^−1^, and 1314 cm^−1^ were mainly associated with fundamental chains of cellulose [15]. The band located at 1046 cm^−1^ was due to the CO stretching of secondary alcohols [35]. The bands at 1110 cm^−1^ and 899 cm^−1^ were due to the presence of β-glucose ether bonds (C-O-C), which were involved with the vibration of the anhydrous pyranose ring skeleton and β-glycosidic bonds between anhydrous glucose rings in cellulose chains [40]. Compared to that of CNF, a new band occurred (at 1476 cm^−1^) in the IR spectra of CCNF, which corresponded to the trimethyl of quaternized amines. This indicated the successful coupling between anhydrous GTMAC and CNF [32].

**Figure 4:**
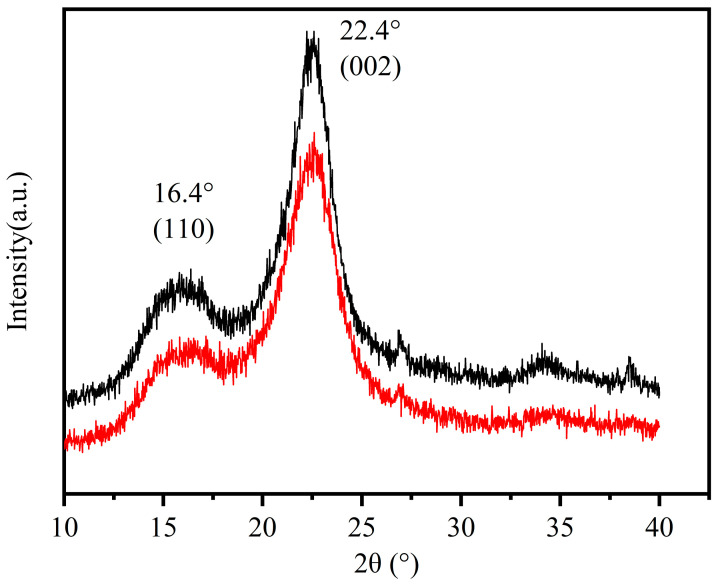
X-ray diffraction (XRD) patterns of CNF (black) and CCNF (red).

XRD analyses of CNF and CCNF are shown in Figure 4. The main indexes were located at 2θ values of approximately 16.4° and 22.4°, which corresponded to the planes of (110), and (002), respectively. This suggested that cellulose I was dominant in the samples [41]. According to the Segal equation [24], the CrI of CNF and CCNF was calculated to be 61% and 62.4%, respectively. The crystallinity degree of CCNF was slightly increased, which was mainly induced by the increased solubilization of the amorphous region in the cellulose during quaternization [32].

### 3.2. Investigation on CR Adsorption on CNF and CCNF

#### 3.2.1. Plotting of Calibration Curves

**Figure 5:**
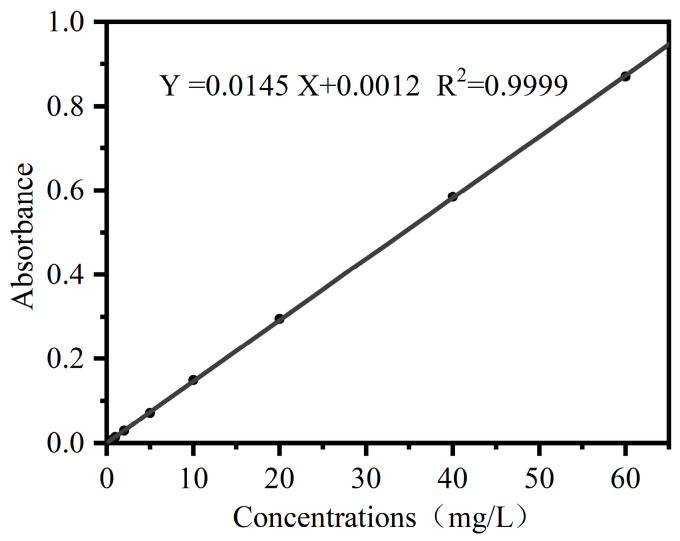
The calibration curves of the absorbance of CR with varying concentrations at 497 nm.

The calibration curves were plotted on the basis of the absorbance of CR with varying concentrations at 497 nm. The results are shown in Figure 5. Linear correlations were found between the CR concentration and its absorbance, with the absorbance between 0~1. The regression equation is as follows: Y = 0.0145X + 0.0012, where X and Y represent the concentration of CR solution and its corresponding absorbance, respectively.

#### 3.2.2. Study on the Adsorption Kinetics of CNF and CCNF

**Figure 6:**
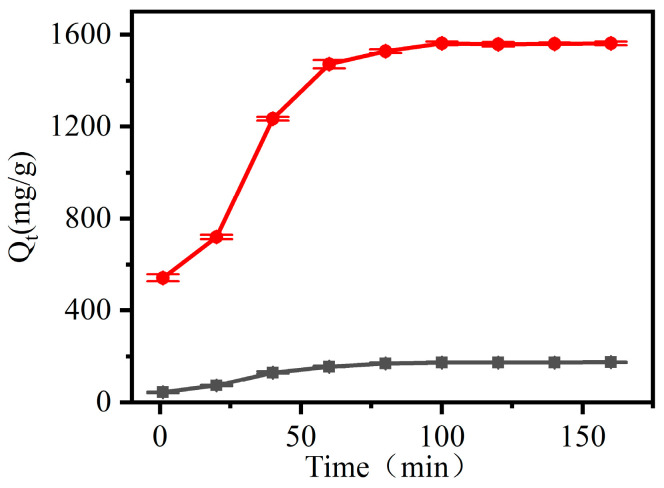
Effect of adsorption time on dye adsorption of CCNF (black) and CCNF (red).

**Figure 7:**
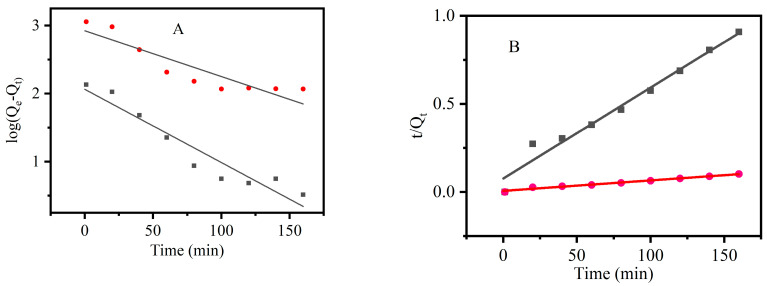
Adsorption kinetics model of CNF (black) and CCNF (red). (**A**): pseudo-first-order kinetics model; (**B**): pseudo-second-order kinetics model.

The effect of adsorption time on the dye adsorption of CNF and CCNF is shown in Figure 6. The Qt increased with an increase in adsorption time, regardless of the material being CNF or CCNF. The adsorption capacity of CNF tended to reach equilibrium after 80 min, whereas that of CCNF became stabilized after 100 min. The experimental data were subjected to fitting using pseudo-first-order and pseudo-second-order kinetics equations, and the results are shown in Figure 7. Relevant parameters of kinetics are shown in Table 1. The pseudo-second-order kinetics model fitting curves of both CNF and CCNF showed higher correlation coefficients R_1_^2^ (0.97561 for CNF and 0.98068 for CCNF) than their pseudo-first-order kinetics model fitting curves with correlation coefficients R_2_^2^ (of 0.91921 for CNF and 0.82491 for CCNF). The *Q_e_* values calculated through the pseudo-first-order kinetics model were 193.80 mg/g (CNF) and 1694.24 mg/g (CCNF). They were close to the corresponding experimental values (181.36 mg/g for CNF and 1678.506 mg/g for CCNF when the initial concentration of CR was 60 mg/L). This indicated that CR adsorption on CNF and CCNF obeyed pseudo-second-order kinetics.

#### 3.2.3. Effect of Initial Concentration of CR on the Adsorption Capacity of CNF and CCNF

**Figure 8:**
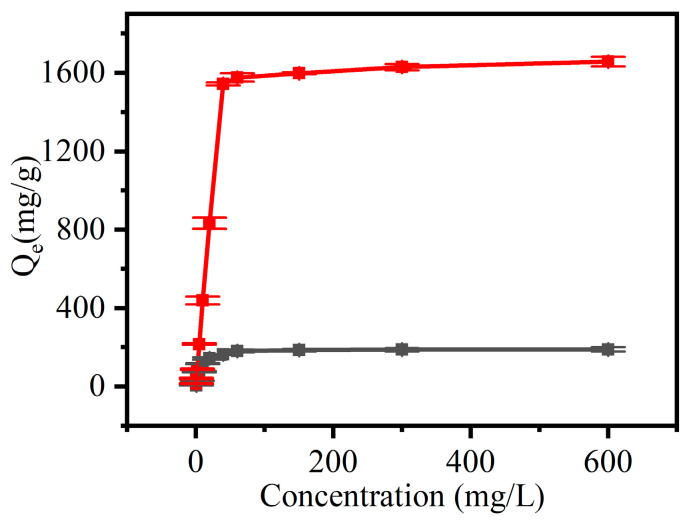
Effect of initial concentration of Congo red (CR) on the adsorption capacity of CNF (black) and cationic CCNF (red).

Figure 8 shows the adsorption on CNF and CCNF of CR with varying initial concentrations. As CR concentration increased from 0.5 mg/L to 600 mg/L, the amount of adsorption on CNF increased from 6.6 mg/g to 190.0 mg/g after adsorption for 100 min, and the corresponding value on CCNF increased from 15.9 mg/g to 1657.9 mg/g. Owing to the driving force induced by the differences in concentration, a higher initial concentration of CR could promote mass transfer, which could increase the adsorption capacity of CNF [42]. When its initial concentration exceeded 40 mg/g, the increase in adsorption capacity became weak, which was attributed to the accumulation of dye molecules that inhibited the adsorption on CNF and CCNF. CCNF presented a significantly higher adsorption capacity than CNF (Figure 8). The adsorption capacity reached 1678.5 mg/g upon equilibrium. It was induced by the presence of abundant positive charges on the CCNF surface after modification, which improved the adsorption of CR dye with divalent anions. Furthermore, the CR adsorption capacity of CCNF prepared from residual Chlorella was higher than that of CCNF prepared from wood pulp (664 mg/g) [32].

#### 3.2.4. Effect of pH Value of CR Solutions on the Adsorption Capacity of CNF and CCNF

**Figure 9:**
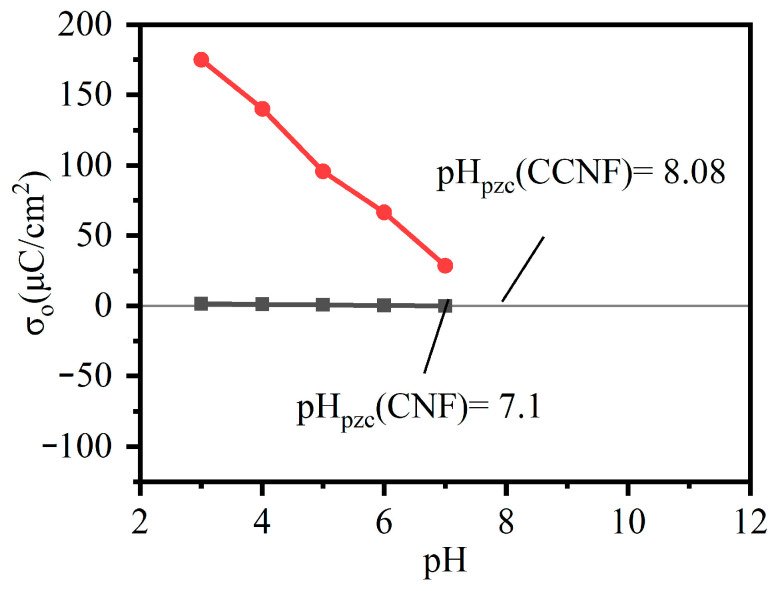
Charge density curves for CNF (black) and CCNF (red).

The pH of the solutions could affect the degree of ionization of ions, thereby affecting the ionic characteristics of adsorbents [43]. Figure 9 represents the charge density versus pH for CNF and CCNF. The pH has a significant effect on the charge density of CCNF, but has little effect on that of CNF. The curves cross the x-axis at their corresponding point of zero charge (pHpzc), which are found to be 7.1, 8.08 for CNF and CCNF. Below pHpzc, CNF and CCNF are positively charged, while at a higher pH, their surfaces are negatively charged.

**Figure 10:**
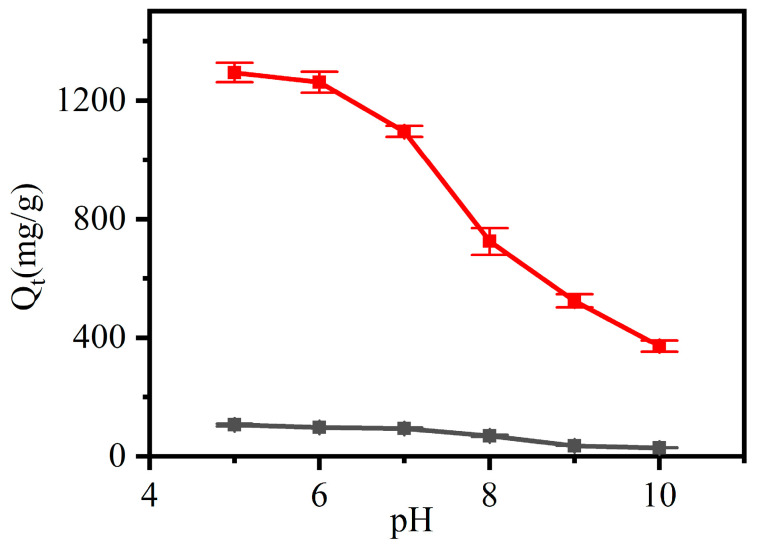
Effect of pH value of Congo red (CR) solutions on the adsorption capacity of CNF (black) and cationic CCNF (red).

**Figure 11:**
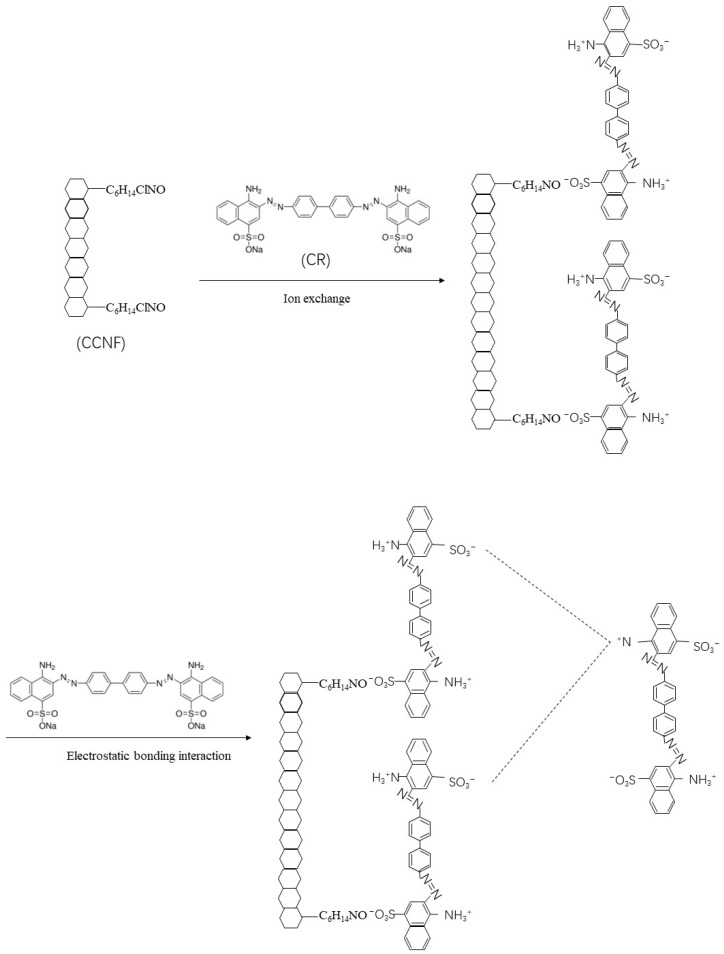
Adsorption of CR by CCNF.

The effect of pH on the adsorption capacity of CNF and CCNF is shown in Figure 10. The effect within the range of pH < 5 is not shown because the color of CR was changed in this range, and CR concentration could not be measured accurately using the UV-Vis spectrometer. The adsorption capacity decreased with the increase in pH from 5 to 10. At a lower pH, the - NH_2_ groups in CR molecules were protonated to -NH_3_^+^ groups. The CR was adsorbed onto CCNF via ion exchange between the quaternary amino groups in the CCNF and the –SO_3_^−^ groups of the CR. The adsorbed CR molecules could further adsorb free CR molecules by electrostatic bonding interaction (Figure 11). As the pH increased, the -NH_3_^+^ group decreased, and the adsorption capacity decreased. When pH > 7, the competition occurring between the excess OH^−^ and the CR anions to interact with the quaternary amino groups of CCNF decreased the adsorption capacity of CCNF [44]. Therefore, the maximum adsorption capacity was achieved for CNF (105.9 mg/g) and CCNF (1293.7 mg/g) when the pH value was 5. In contrast to CNF, CCNF showed greater adsorption capacity at all pH values. This implied that positive ions on the surface of CCNF improved its adsorption capacity for negatively charged dyes.

#### 3.2.5. Adsorption Isotherms

**Figure 12:**
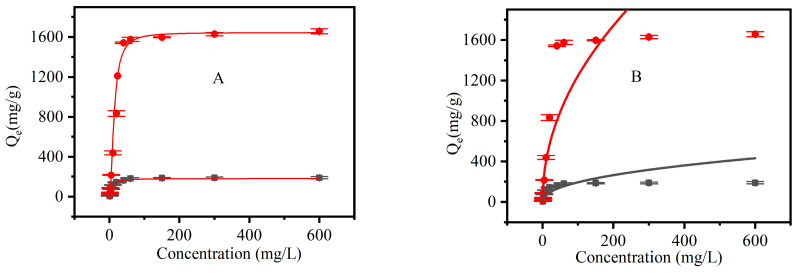
Adsorption isotherms of CNF (black) and CCNF (red). (**A**): Langmuir isotherm model; (**B**): Freundlich isotherm model.

Langmuir and Freundlich isotherm model equations were used to perform fittings for the adsorption data at different initial concentrations of CR. This helped to determine the interactions between adsorbents and dye molecules and further describe the dye adsorption process. The fitting results are shown in Figure 12, and the corresponding correlation coefficients are listed in Table 2. Compared with those of the Freundlich isotherm model, the maximum adsorption values (*Q_m_*) calculated via the Langmuir model (180.97 mg/g and 1645.2 mg/g at the initial concentration of dye of 600 mg/L) were closer to experimental values (190.0 mg/g and 1657.89 mg/g, respectively). Furthermore, the fitting curves of the Langmuir model showed correlation coefficients (R^2^) of 0.99926 and 0.99543, both of which were higher than those of the Freundlich model (0.8192 and 0.8609, respectively). Therefore, the Langmuir model fitted the experimental data better. Meanwhile, CNF and CCNF had homogenous surfaces; therefore, no significant changes were observed in the adsorption capacity at different locations on the surface, which was characterized by monolayer adsorption.

## 4. Conclusions

In this study, the nanofiber of cellulose was prepared from the waste of Chlorella residue through high-pressure homogenization, which was subjected to modifications by quaternization. Furthermore, the adsorption capacity of CNF and CCNF for negatively charged CR dye was evaluated. The CR adsorption of CNF and CCNF followed the pseudo-second-order kinetics model. The adsorption capacity was saturated after CNF and CCNF had contact with CR for 100 min. CCNF showed considerably higher adsorption capacity than CNF. The initial concentration of CR had a pronounced effect on the adsorption capacity of CNF and CCNF. When the initial concentration of CR was below 40 mg/g, the adsorption capacity of CNF and CCNF was considerably increased with the increasing initial concentration. As shown by the adsorption isotherm analysis of CNF and CCNF, the Langmuir model fitted best with the experimental data, which could better explain the adsorption capacity of CNF and CCNF. pH had a great effect on the CR adsorption of CNF and CCNF, and the acidic environment was favorable for CR adsorption. The effect of pH was especially distinct for CCNF. In this study, CCNF presented a more advantageous adsorption capacity with a maximum value of 1657.89 mg/g, which was 8.7 times higher than that of CNF. It indicated that positive ions on the surface of CCNF increased its adsorption capacity for negatively charged dye. 

This work was performed to investigate the reuse of Chlorella waste for the production of CNF, which was used for dye adsorption. Given its superior properties, the CCNF derived from the reuse of Chlorella waste represents a promising environmentally friendly adsorbent candidate for the removal of anionic dyes from industrial wastewater.

## Figures and Tables

**Table 1:** Kinetic parameters of Congo red (CR) adsorption on CNF and CCNF. Determined using different models.

	Pseudo-First-Order Kinetics Model			Pseudo-Second-Order Kinetics Model		
	*Q_e_* (mg/g)	*K*_1_ (min^−1^)	R_1_^2^	*Q_e_* (mg/g)	*K*_2_ (g/mg·min)	R_2_^2^
CCNF	836.97	0.01545	0.82491	1694.24	3.483 × 10^–7^	0.98068
CNF	115.82	0.02478	0.91921	193.80	3.337 × 10^–4^	0.97561

**Table 2:** Parameters of different isotherm models applied to Congo red (CR) adsorption of cellulose nanofiber (CNF) and cationic CNF (CCNF).

	Langmuir Isotherm Constants			Freundlich Isotherm Constants		
	*K*_L_ (mg/L)	*Q_m_* (mg/g)	R^2^	P (mg/g)	N	R^2^
CNF	0.08377	180.97	0.99926	25.19174	2.25	0.8192
CCNF	0.00803	1645.26	0.99543	135.30359	2.07	0.8609

## Data Availability

Any further details relevant to this study may be obtained from the authors upon reasonable request.
